# Using a new diagnostic tool to predict lymph node metastasis in advanced epithelial ovarian cancer leads to simple lymphadenectomy decision rules: A multicentre study from the FRANCOGYN group

**DOI:** 10.1371/journal.pone.0258783

**Published:** 2021-10-19

**Authors:** Camille Mimoun, Xavier Paoletti, Thomas Gaillard, Adrien Crestani, Jean-Louis Benifla, Matthieu Mezzadri, Sofiane Bendifallah, Cyril Touboul, Alexandre Bricou, Yohann Dabi, Geoffroy Canlorbe, Yohan Kerbage, Vincent Lavoué, Lobna Ouldamer, Lise Lecointre, Charles Coutant, Arnaud Fauconnier, Roman Rouzier, Cyrille Huchon

**Affiliations:** 1 Department of Gynecology and Obstetrics, Lariboisière University Hospital, AP-HP, Paris, France; 2 Research Unit EA 7285 "Risk and Safety in Clinical Medicine for Women and Perinatal Health", UVSQ, Montigny-Le-Bretonneux, France; 3 Department of Surgical Oncology, Curie Institute, Saint-Cloud, France; 4 INSERM U900 STAMPM Team, Saint Cloud, France; 5 Department of Gynecology and Obstetrics, Tenon University Hospital, AP-HP, Paris, France; 6 Department of Obstetrics, Gynecology and Reproductive Medicine, CH Jean Verdier, AP-HP, Bondy, France; 7 Department of Gynecology and Obstetrics, CHIC, Créteil, France; 8 Department of Gynecological and Breast Surgery and Oncology, Pitié-Salpêtrière University Hospital, AP-HP, Paris, France; 9 Department of Gynecologic Surgery, Jeanne de Flandre Hospital, CHU of Lille, Loos, France; 10 Department of Gynecology, CHU de Rennes, Rennes, France; 11 Department of Obstetrics and Gynecology, Bretonneau Hospital, CHU of Tours, Tours, France; 12 Department of Obstetrics and Gynecology, University Hospital Center, Strasbourg, France; 13 Department of Surgical Oncology, Georges-François Leclerc Cancer Center, Dijon, France; 14 Department of Obstetrics and Gynecology, Poissy-St Germain Hospital, Poissy, France; Universitá Sapienza di Roma, ITALY

## Abstract

**Objective:**

The aim of this study was to develop a new diagnostic tool to predict lymph node metastasis (LNM) in patients with advanced epithelial ovarian cancer undergoing primary cytoreductive surgery.

**Materials and method:**

The FRANCOGYN group’s multicenter retrospective ovarian cancer cohort furnished the patient population on which we developed a logistic regression model. The prediction model equation enabled us to create LNM risk groups with simple lymphadenectomy decision rules associated with a user-friendly free interactive web application called shinyLNM.

**Results:**

277 patients from the FRANCOGYN cohort were included; 115 with no LNM and 162 with LNM. Three variables were independently and significantly (p<0.05) associated with LNM in multivariate analysis: pelvic and/or para-aortic LNM on CT and/or PET/CT (p<0.00), initial PCI ≥ 10 and/or diaphragmatic carcinosis (p = 0.02), and initial CA125 ≥ 500 (p = 0.02). The ROC-AUC of this prediction model after leave-one-out cross-validation was 0.72. There was no difference between the predicted and the observed probabilities of LNM (p = 0.09). Specificity for the group at high risk of LNM was 83.5%, the LR+ was 2.73, and the observed probability of LNM was 79.3%; sensitivity for the group at low-risk of LNM was 92.0%, the LR- was 0.24, and the observed probability of LNM was 25.0%.

**Conclusion:**

This new tool may prove useful for improving surgical planning and provide useful information for patients.

## Introduction

Ovarian cancer is the second most common gynecological cancer in the United States with an expected estimated 21 750 new cases and 13 940 deaths in 2020 [1]. The backbone treatment for advanced epithelial ovarian cancer (AEOC) associates complete cytoreductive surgery with platinum- and taxane-based chemotherapy [2–4].

The conception of AEOC surgery has changed recently, in particular for the controversial question of pelvic and para-aortic lymphadenectomy [5–9]. The LION trial was the first prospective randomized trial to compare systematic lymphadenectomy with no lymphadenectomy during macroscopically complete primary resection of patients with “no suspect lymph node”. Lymphadenectomy was not associated with longer overall or progression-free survival than the no-lymphadenectomy, but it was associated with relatively high morbidity and mortality [6].

Today, therefore, the challenge is to triage patients appropriately, to distinguish those with “no suspect lymph node” who should not have a lymphadenectomy from those with “suspect lymph nodes” who should have lymphadenectomy. Two effective diagnostic tools currently exist: preoperative imagery and intraoperative clinical evaluation, which have respectively a specificity of 85% and 83.6% and a sensitivity of 79% and 62.5%, for the prediction of lymph node metastasis (LNM) in AEOC [10–12]. No other diagnostic tool exists nowadays to predict LNM in AEOC.

The aim of this study was to develop a new diagnostic tool to predict pelvic and/or para-aortic LNM and risk groups leading to simple lymphadenectomy decision rules in patients with AEOC undergoing primary cytoreductive surgery.

## Materials and method

### Study design and population

The study population was extracted from the ovarian cancer database of the FRANCOGYN study group, a retrospective multicentric cohort from 11 referral centers in France (Tenon, Jean Verdier, Créteil, Poissy, La Pitié Salpêtrière, Lariboisière, Lille, Rennes, Tours, Strasbourg and Dijon) including all patients managed for ovarian cancer from January 2000 through December 2017.

This study reviewed records of all consecutive patients who underwent surgery and had histologically confirmed AEOC of stages IIB to IV according to the FIGO classification, but included only those considered suitable for primary complete resection of their disease on initial assessment and who underwent pelvic and/or paraaortic lymphadenectomy with the removal of 10 or more lymph nodes.

The Ethics Committee for Research in Obstetrics and Gynecology approved the research protocol (CEROG 2019-GYN-605). All the data were fully anonymized. As per French law, the requirement for informed consent was waived for this type of study that used only de-identified data gained from clinical practice.

### Gold standard

Histology was the gold standard used to diagnose pelvic and/or paraaortic LNM. The total number of lymph nodes removed, the number of positive lymph nodes and the number of negative lymph nodes was notified. Specialized pathologists reviewed all removed lymph nodes.

### Surgical procedure

No patients received chemotherapy before surgery. Surgery included at least hysterectomy, bilateral salpingo-oophorectomy, omentectomy, pelvic and/or para-aortic lymphadenectomy and removal of any other intraperitoneal metastasis. Surgical staging followed the FIGO staging system. Disease extent at the start of each surgical procedure was quantified with the peritoneal cancer index (PCI), as described by Sugarbaker [13]. The surgery was classified as complete resection (CC0) when all visible tumor was removed (no macroscopic residual tumor) at the end of the intervention, CC1 when it was ≤ 2.5 mm and CC2 when it was more than 2.5 mm but less than 2.5 cm. Gynecologic oncology specialists performed all cytoreductive surgeries.

### Data collection

The following clinical and paraclinical items were collected: age at diagnosis, body mass index (BMI), personal or family history of gynecological cancers, presence or absence of identified genetic mutations, the American society of anesthesiologists (ASA) score [14], preoperative CA125, preoperative radiological characteristics (computed tomography (CT) and positron tomography emission/ computed tomography (PET/CT)). The tumor histology was detailed: histological type and tumor grade.

### Statistical analysis

We compared patients with no LNM to patients with LNM. We carried out univariate analysis using a quantitative (Student’s t-test) or a qualitative (Chi^2^ test) test as appropriate. Some quantitative variables were dichotomized to maximize the accuracy value. The accuracy of each variable for the prediction of LNM was assessed on the basis of sensitivity, specificity, positive likelihood ratio (LR+) and negative likelihood ratio (LR-) and diagnostic odds ratio (DOR). Variables associated with LNM in the univariate analysis at a threshold of p<0.20 were selected for the multivariate analysis.

The multiple logistic regression analysis, performed with a backward procedure, was used to estimate the most predictive combination of variables that was independently associated with LNM (p<0.05). Adjusted DORs (aDOR) were calculated. Missing data were treated as a distinct category.

The predictive accuracy of the model was assessed in terms of its discrimination and calibration. Discrimination is the ability to differentiate patients with no LNM from patients with LNM. It was studied using the receiver operating characteristic (ROC) curve and summarized by the area under the curve (AUC) [15]. Calibration is the agreement between the observed outcome frequencies and the predicted probabilities. It was studied using graphical representation of the relationship between these two results (calibration curve). We also evaluated the average and maximal errors between the prediction and observation, obtained from the calibration curve.

Internal validation of the prediction model used leave-one-out cross-validation to correct for overoptimism in the predictive performance of the model [16]. This method consists of splitting the data set randomly into n partitions. For each of the n-th iterations, n − 1 partitions served as the training set and the left-out sample as the test set [17].

We created risk groups of pelvic and/or para-aortic LNM by choosing threshold values of the prediction model equation that maximised classification rates [18]. From this, we proposed simple lymphadenectomy decision rules associated with a user-friendly free interactive web app, called shinyLNM, that determines the risk group and their predicted probability of LNM for individual patients.

Differences were considered significant at a level of p<0.05. Statistical analyses were performed using STATA 13.0 (Stata Corp.; College Station, TX, USA). The shinyLNM app was programmed with the package Shiny from R Studio.

## Results

### Characteristics of study population

**Fig 1:**
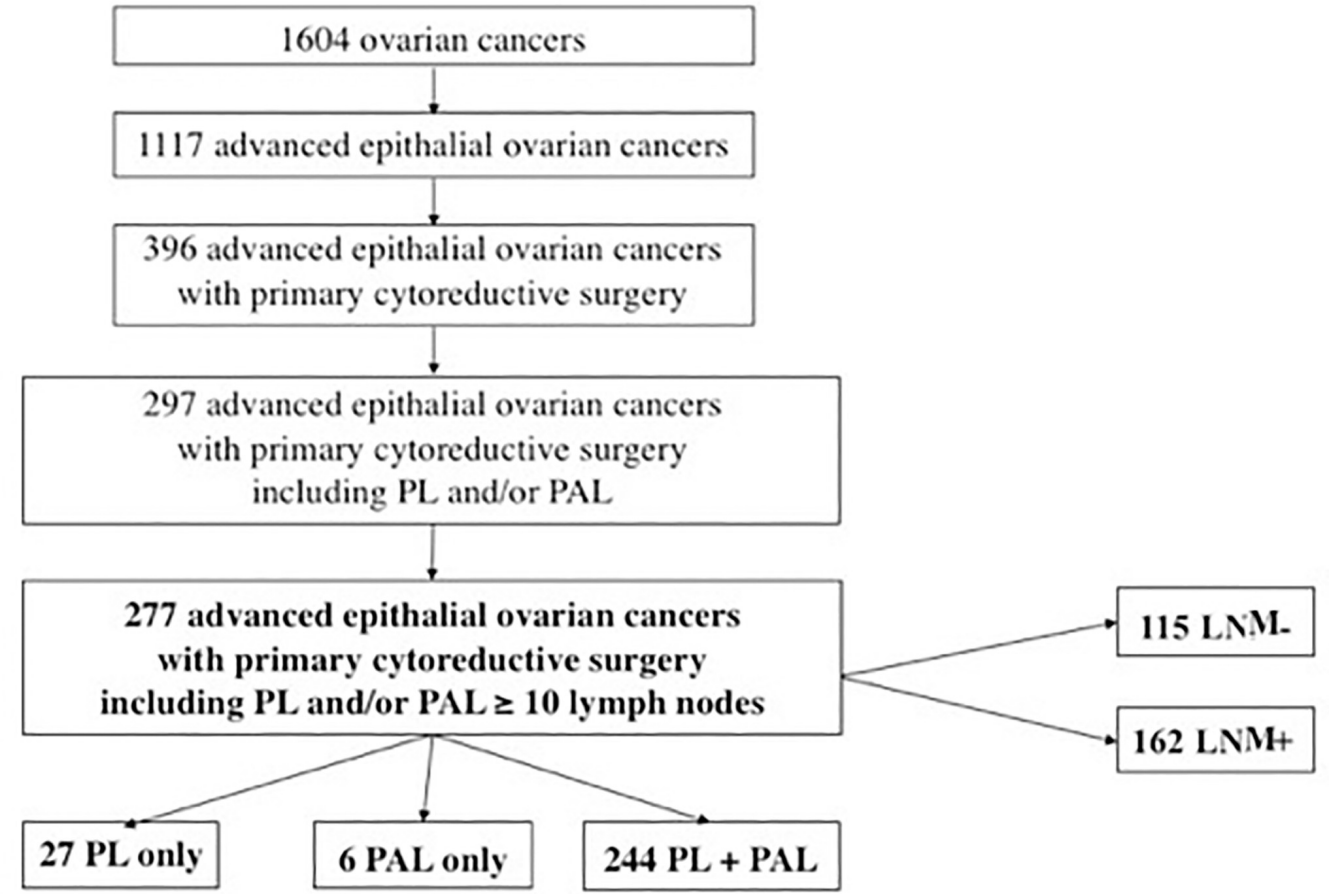
Flow chart. PL: pelvic lymphadenectomy; PAL: para-aortic lymphadenectomy; LNM: lymph node metastasis.

Fig 1 presents the flow chart of the study population: 277 patients from the FRANCOGYN cohort, 115 with no LNM and 162 with LNM. This population’s characteristics are presented in Table 1. These two groups did not differ statistically except for FIGO stage (p<0.001), initial CA125 (p<0.001), initial PCI (p<0.001), bowel resection (p<0.001), and duration of surgery (p = 0.01).

**Table 1:** Clinical, tumor, biological and surgical characteristics of the population.

Variables	All Population	LNM -	LNM +
	N = 277	n = 115	n = 162
	mean +/- SD	mean +/- SD	mean +/- SD
	or n (%)^a^	or n (%)^a^	or n (%)^a^
***CLINICAL CHARACTERISTICS***			
**Age** (years)	57.4 +/- 11.3	58.04 +/- 10.9	56.9 +/- 11.6
**BMI** (kg/m^-2^)	24.5 +/- 5.8	23.8 +/- 4.3	25.0 +/- 5.8
**Gravidity**	1.9 +/- 1.4	2.1 +/- 1.5	1.8 +/- 1.4
**Parity**	1.7 +/- 1.4	1.8 +/- 1.4	1.7 +/- 1.4
**Menopause**			
• No	64 (24.9)	29 (26.6)	35 (23.7)
• Yes	193 (75.1)	80 (73.4)	113 (76.3)
**Mutation**			
• No mutation	39 (45.2)	20 (64.5)	19 (46.3)
• BRCA1	22 (30.6)	7 (22.6)	15 (36.6)
• BRCA2	9 (12.5)	4 (12.9)	5 (12.2)
• Others	2 (2.8)	0 (0.0)	2 (4.9)
**ASA score**			
• 0	2 (1.0)	0 (0.0)	2 (1.8)
• 1	121 (63.0)	53 (66.3)	68 (60.7)
• 2	54 (28.1)	22 (27.5)	32 (28.6)
• 3	15 (7.8)	5 (6.3)	10 (3.9)
**Previous surgery**			
• No	41 (35.3)	19 (40.4)	22 (31.9)
• Yes	75 (64.7)	28 (59.6)	47 (68.1)
***TUMOR CHARACTERISTICS***			
**FIGO stage**			
• 2B-2C	21 (7.6)	20 (17.4)	1 (0.6)
• 3A-3B-3C	228 (82.3)	81 (70.4)	147 (90.7)
• 4A-4B	28 (10.1)	14 (12 .2)	14 (8.6)
**Tumor histological type**			
• Serous	214 (77.3)	86 (74.8)	128 (79.0)
• Endometrioid	37 (13.4)	18 (15.7)	19 (11.7)
• Mucinous	7 (2.5)	4 (3.5)	3 (1.9)
• Clear-cell carcinoma	15 (5.4)	4 (3.5)	11 (6.8)
• Transitional-cell carcinoma	4 (1.4)	3 (2.6)	1 (0.6)
***BIOLOGICAL CHARACTERISTICS***			
**Initial CA125 level** (IU/L)	957.6 +/- 1677.3	597.9 +/- 926.5	1178.5 +/- 1974.6
***SURGICAL CHARACTERISTICS***			
**Initial PCI score**	7.9 +/- 9.4	4.1 +/- 5.2	10.6 +/- 10.7
**Residual disease after surgery**			
• No visible tumor, complete cytoreduction	252 (92.3)	106 (93.8)	146 (91.8)
• Tumor nodules ≤ 2.5 cm	12 (4.4)	3 (2.7)	9 (5.7)
• Tumor nodules > 2.5 cm	8 (2.9)	4 (3.5)	4 (2.5)
**Bowel resection**			
• No	159 (57.4)	83 (72.2)	76 (46.9)
• Yes, without ileostomy or colostomy	96 (34.7)	27 (23.5)	69 (42.6)
• Yes, with ileostomy or colostomy	22 (7.9)	5 (4.4)	17 (10.5)
**Estimated blood loss** (ml)	931.3 +/- 783.4	842.4 +/- 803.1	994.1 +/- 772.9
**Transfusion**			
• No	37 (41.6)	20 (50.0)	17 (34.7)
• Yes	52 (58.4)	20 (50.0)	32 (65.3)
**Duration of surgery** (minutes)	381.5 +/- 145.2	331.6 +/- 102.6	414. 5 +/- 160.1

* Student’s test.

** Chi2‘s test.

a: percentages are calculated on the total number of patients despite some missing values.

LNM: Lymph Node Metastasis; SD: Standard Derivation; BMI: Body Mass Index; BRCA: BReast CAncer; ASA: American Society of Anaesthesiologists; FIGO: Fédération Internationale de Gynécologie-Obstétrique; CA: Carcinome Antigen; PCI: Peritoneal Carcinomatosis Index.

### Prediction model and risk groups

The findings from the univariate analysis are presented in Table 2.

**Table 2:** Univariate analysis for predicting lymph node metastasis.

Variables	Total, n/N	LNM +, n/N	LNM -, n/N	Se (%)	Sp (%)	LR+	LR-	DOR 95% CI	p*
***CLINICAL CHARACTERISTICS***									
Age ≥ 50 years	211/277	120/277	91/277	74.1	20.9	0.94	1.24	0.75 0.42–1.34	0.33
Menopause	193/257	113/257	80/257	76.4	26.6	1.04	0.89	1.17 0.66–2.07	0.59
BRCA mutation	33/72	22/72	11/72	53.7	64.5	1.51	0.72	2.11 0.79–5.62	**0.13**
***TUMOR CHARACTERISTICS***									
Serous histological type	214/277	128/277	86/277	79.0	25.2	1.06	0.83	1.27 0.72–2.24	0.41
***RADIOLOGICAL CHARACTERISTICS*: CT**									
Involvement of 1 ovary	120/186	68/186	52/186	61.3	30.7	0.88	1.26	0.70 0.37–1.31	0.26
Involvement of 2 ovaries	62/133	42/133	20/133	50.6	60.0	1.27	0.82	1.54 0.75–3.15	0.24
Involvement of 1 and/or 2 ovary(ies)	182/190	110/190	72/190	97.3	6.5	1.04	0.41	2.55 0.58–11.10	**0.20**
Diaphragmatic carcinosis	20/172	12/172	8/172	12.0	88.9	1.08	0.99	1.09 0.42–2.83	0.85
Omentum carcinosis	42/173	26/173	16/173	25.5	77.5	1.13	0.96	1.18 0.57–2.40	0.66
Small intestine involvement	4/142	4/142	0/142	4.7	100.0	-	0.95	-	**0.10**
Colon involvement	26/173	19/173	7/173	18.4	90.0	1.84	0.91	2.04 0.80–5.19	**0.13**
Bowel involvement	26/177	19/177	7/177	18.1	90.3	1.86	0.91	2.05 0.81–5.22	**0.12**
Liver metastasis	7/171	3/171	4/171	3.0	94.4	0.53	1.03	0.52 0.11–2.41	0.39
Ascites	85/158	51/158	34/158	52.6	44.3	0.94	1.07	0.88 0.46–1.68	0.70
Pelvic LNM	51/175	39/175	12/175	37.1	82.9	2.17	0.76	2.86 1.34–6.09	**0.01**
Para-aortic LNM	41/141	31/141	10/141	35.6	81.5	1.92	0.79	2.44 1.06–5.60	**0.03**
Pelvis and/or Para-aortic LNM	61/175	48/175	13/175	45.7	81.4	2.46	0.67	3.69 1.75–7.79	**0.00**
Supra-diaphragmatic LNM	11/126	8/126	3/126	9.9	93.3	1.48	0.97	1.53 0.38–6.15	0.54
Pleural effusion	14/178	9/178	5/178	8.4	93.0	1.19	0.99	1.21 0.39–3.79	0.74
***RADIOLOGICAL CHARACTERISTICS*: PET/CT**									
Involvement of 1 ovary	21/30	12/30	9/30	66.7	25.0	0.89	1.33	0.67 0.13–3.53	0.63
Involvement of 2 ovary	9/30	7/30	2/30	38.9	83.3	2.33	0.73	3.18 0.49–20.72	**0.20**
Involvement of 1 and/or 2 ovary(ies)	26/30	16/30	10/30	88.9	16.7	1.07	0.67	1.60 0.19–13.82	0.67
Diaphragmatic carcinosis	4/31	3/31	1/31	15.8	91.7	1.89	0.92	2.06 0.18–23.75	0.55
Omentum carcinosis	4/32	4/32	0/32	20.0	100.0	-	0.80	-	**0.10**
Small intestine involvement	1/32	0/32	1/32	0.0	91.7	0.00	1.09	-	**0.20**
Colon involvement	5/32	3/32	2/32	15.0	83.3	0.90	1.02	0.88 0.12–6.42	0.90
Bowel involvment	6/32	3/32	3/32	15.0	75.0	0.60	1.13	0.53 0.08–3.32	0.49
Liver metastasis	1/32	1/32	0/32	5.0	100.0	-	0.95	-	0.44
Ascites	2/32	1/32	1/32	5.0	91.7	0.60	1.04	0.58 0.03–10.76	0.71
Pelvic LNM	8/32	8/32	0/32	40.0	100.0	-	0.60	-	**0.01**
Para-aortic LNM	10/33	8/33	2/33	38.1	83.3	2.29	0.74	3.08 0.49–19.16	**0.20**
Pelvis and/or Para-aortic LNM	14/33	12/33	2/33	57.1	83.3	3.43	0.51	6.67 0.97–45.94	**0.03**
Supra-diaphragmatic LNM	4/33	3/33	1/33	14.3	91.7	1.71	0.94	1.83 0.16–20.84	0.62
Pleural effusion	1//33	1/33	0/33	4.8	100.0	-	0.95	-	0.45
***RADIOLOGICAL CHARACTERISTICS*: CT and/or PET/CT**									
Involvement of 1 ovary	126/192	72/192	54/192	62.6	29.9	0.89	1.25	0.71 0.38–1.33	0.28
Involvement of 2 ovaries	66/146	45/146	21/146	50.0	62.5	1.33	0.80	1.67 0.84–3.31	**0.14**
Involvement of 1 and/or 2 ovary(ies)	188/196	114/196	74/196	97.4	6.33	1.04	0.41	2.57 0.59–11.18	**0.19**
Diaphragmatic carcinosis	21/172	13/172	8/172	13.0	88.9	1.17	0.98	1.20 0.47–3.06	0.71
Omentum carcinosis	45/177	29/177	16/177	27.9	78.1	1.27	0.92	1.38 0.68–2.79	0.37
Small intestine involvement	5/155	4/155	1/155	4.3	98.4	2.67	0.97	2.74 0.30–26.46	0.36
Colon involvement	31/179	22/179	9/179	20.6	87.5	1.64	0.91	1.81 0.78–4.23	**0.16**
Bowel involvment	32/183	22/183	10/183	20.2	86.5	1.49	0.92	1.62 0.71–3.67	0.25
Liver metastasis	8/177	4/177	4/177	3.9	94.5	0.70	1.02	0.69 0.17–2.87	0.61
Ascites	85/171	51/171	34/171	49.0	49.3	0.97	1.03	0.93 0.50–1.73	0.83
Pelvic LNM	56/182	44/182	12/182	40.4	83.6	2.46	0.71	3.44 1.62–7.32	**0.001**
Para-aortic LNM	50/155	38/155	12/155	40.4	80.3	2.05	0.74	2.77 1.28–6.01	**0.01**
Pelvis and/or Para-aortic LNM	70/182	56/182	14/182	51.4	80.8	2.68	0.60	4.45 2.14–9.29	**0.00**
Supra-diaphragmatic LNM	14/140	10/140	4/140	11.4	92.3	1.48	0.96	1.54 0.45–5.21	0.49
Pleural effusion	14/184	9/184	5/184	8.1	93.2	1.18	0.99	1.20 0.38–3.75	0.75
***BIOLOGICAL CHARACTERISTICS***									
Initial CA125 level ≥ 500	105/247	77/247	28/247	50.3	70.2	1.69	0.71	2.39 1.37–4.17	**0.00**
***SURGICAL CHARACTERISTICS***									
Initial PCI score ≥ 10	24/77	20/77	4/77	44.4	87.5	3.56	0.64	5.60 1.55–20.26	**0.00**
Ascites	90/161	58/161	32/161	59.8	50.0	1.20	0.80	1.49 0.78–2.82	0.22
Omental cake	63/150	48/150	15/150	51.6	73.7	1.96	0.66	2.99 1.42–6.27	**0.00**
Peritoneal carcinosis	84/152	59/152	25/152	62.1	56.1	1.42	0.68	2.10 1.06–4.14	**0.03**
Diaphragmatic carcinosis	55/150	40/150	15/150	43.0	73.7	1.63	0.77	2.11 1.01–4.39	**0.04**
Stomach infiltration	6/146	4/146	2/146	4.4	96.4	1.24	0.99	1.26 0.22–7.14	0.80
Mesenteric retraction	11/146	6/146	5/146	6.6	90.9	0.73	1.03	0.71 0.20–2.45	0.58
Bowel infiltration	65/153	49/153	16/153	51.6	72.4	1.87	0.67	2.80 1.35–5.77	**0.00**
Liver metastasis	10/150	7/150	3/150	7.6	94.8	1.47	0.97	1.51 0.37–6.13	0.56
Initial PCI score ≥ 10 and/or Diaphragmatic carcinosis	66/166	49/166	17/166	48.0	73.4	1.81	0.71	2.56 1.28–5.12	**0.01**

* Chi2 test.

LNM: Lymph Node Metastasis; CI: Confidence Interval; DOR: Diagnostic Odd Ratio; Se: Sensibility; Sp: Specificity; LR -: negative Likelihood Ratio; LR +: positive Likelihood Ratio; CT: Computed Tomography; PET/CT: Positron Emission Tomography/Computed Tomography; FIGO: Fédération Internationale de Gynécologie-Obstétrique; CA: Carcinome Antigen; PCI: Peritoneal Carcinomatosis Index.

The multiple logistic regression analysis identified three variables independently and significantly (p<0.05) associated with pelvic and/or para-aortic LNM: pelvic and/or para-aortic LNM on CT and/or PET/CT (aDOR = 5.02 95%CI [2.42–10.44], p<0.001), initial PCI ≥ 10 and/or diaphragmatic carcinosis (aDOR = 2.34 95%CI [1.13–4.83], p = 0.02), and initial CA125 ≥ 500 (aDOR = 2.03 95%CI [1.14–3.61], p = 0.02) (Table 3).

**Table 3:** Prediction model.

Variables	aDOR	95% CI	p
**Pelvic and/or Para-aortic LNM on CT and/or PET/CT**			
No	1		**0.00**
**Yes**	**5.02**	2.42–10.44	
**Initial PCI score ≥ 10 and/or diaphragmatic carcinosis**			
No	1		**0.02**
**Yes**	**2.34**	1.13–4.83	
**Initial PCI CA125 level ≥ 500**			**0.02**
No	1		
**Yes**	**2.03**	1.14–3.61	

aDOR: adjusted Odd Ratio; CI: Confidence Interval; LNM: Lymph Node Metastasis; CT: Computed Tomography; PET/CT: Positron Emission Tomography/Computed Tomography; CA: Carcinome Antigen; PCI: Peritoneal Carcinomatosis Index.

**Fig 2:**
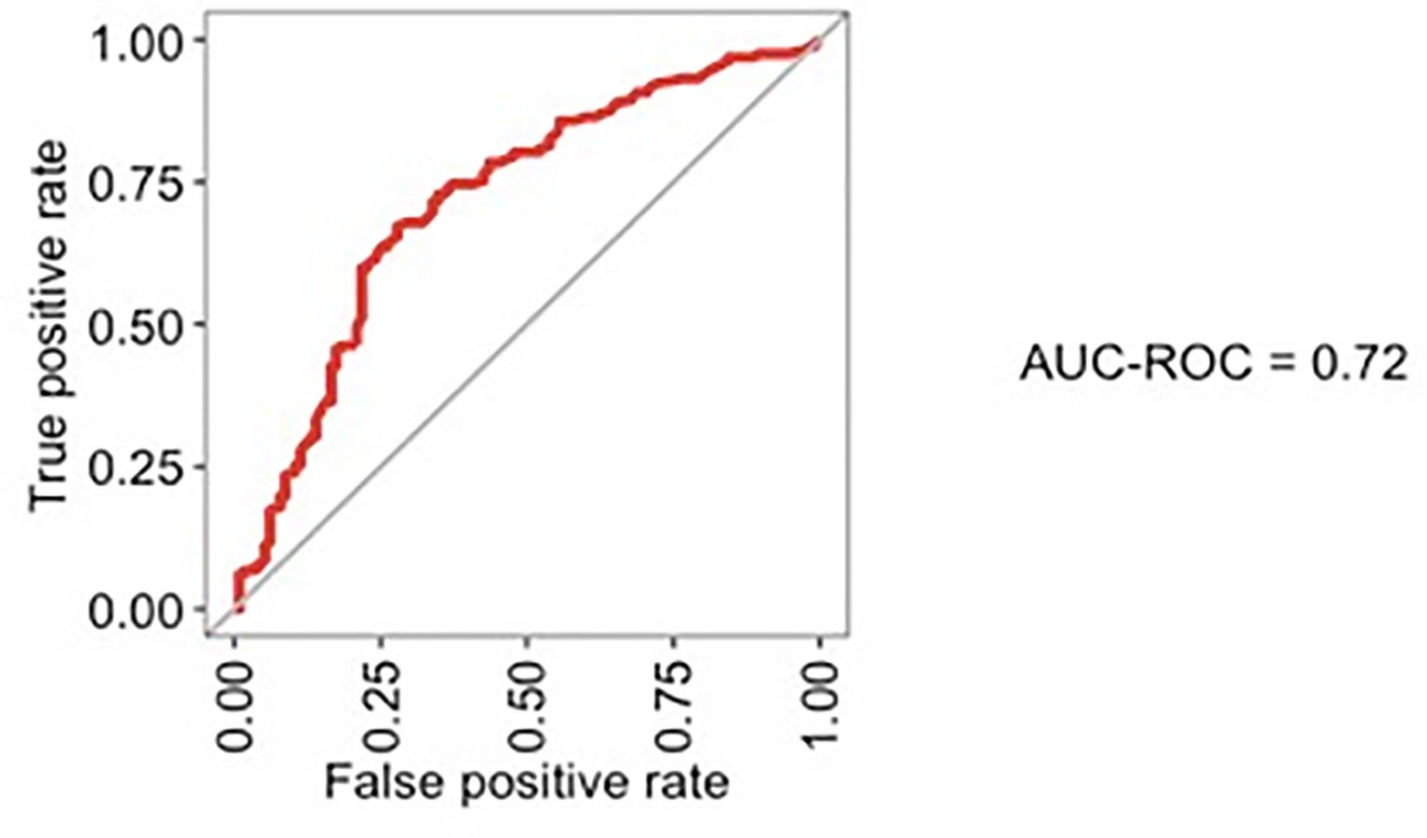
ROC curve of the logistic regression model and performance after leave-one-out cross-validation. ROC: Receiving Operating Curve; AUC: Area Under the Curve.

**Fig 3:**
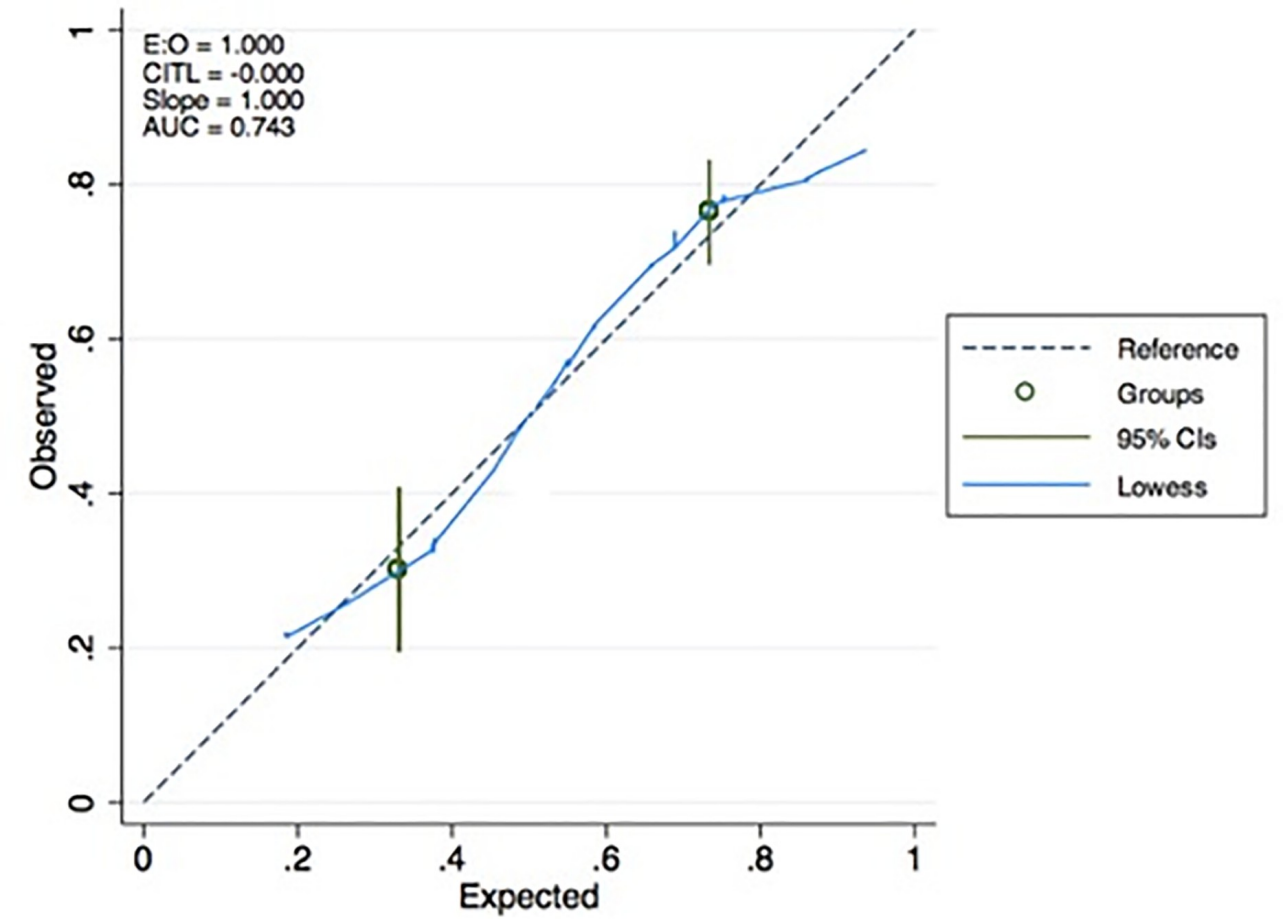
Internal calibration of the logistic regression model to predict lymph node positivity. The horizontal axis represents the predicted probability of LNM and the vertical axis its actual probability. Perfect prediction would correspond to the 45 degrees broken line. The solid blue line indicates the observed (apparent) logistic regression model performance. Circles correspond to the to the risk groups of predicted probability for LNM with their 95%CI. There was no difference between the predicted probabilities and the observed rates of LNM (p = 0.09). AUC: area under the curve; CITL: calibration in the large.

The ROC-AUC of this prediction model after leave-one-out cross-validation was 0.72 (Fig 2). The predicted and the observed probabilities of LNM, shown in the calibration curve in Fig 3, did not differ significantly (p = 0.09).

We created risk groups of pelvic and/or para-aortic LNM based on the prediction model equation, using the following coding variables.

- LNM: pelvic and/or para-aortic LNM on CT and/or PET/CT, no = 0, yes = 1
- PCI: initial PCI ≥ 10 and/or diaphragmatic carcinosis, no = 0, yes = 1
- CA125: initial CA125 ≥ 500, no = 0, yes = 1

$$Pr=\frac{1}{1+e^{-(-0.51–1.61\times LNM–0.85\times PCI–0.71\times CA125)}}$$

- the low-risk group was defined for a probability < 0.377, its sensibility for the prediction of LNM was 92.0% and its LR- was 0.24; in the low-risk group, the observed probability of LNM was 25.0%;
- the high-risk group was defined for a probability ≥ 0.740, its specificity for the prediction of LNM was 83.5% and its LR+ was 2.73; in the high-risk group the observed probability of LNM was 79.3%.

### Clinical utility

**Fig 4:**
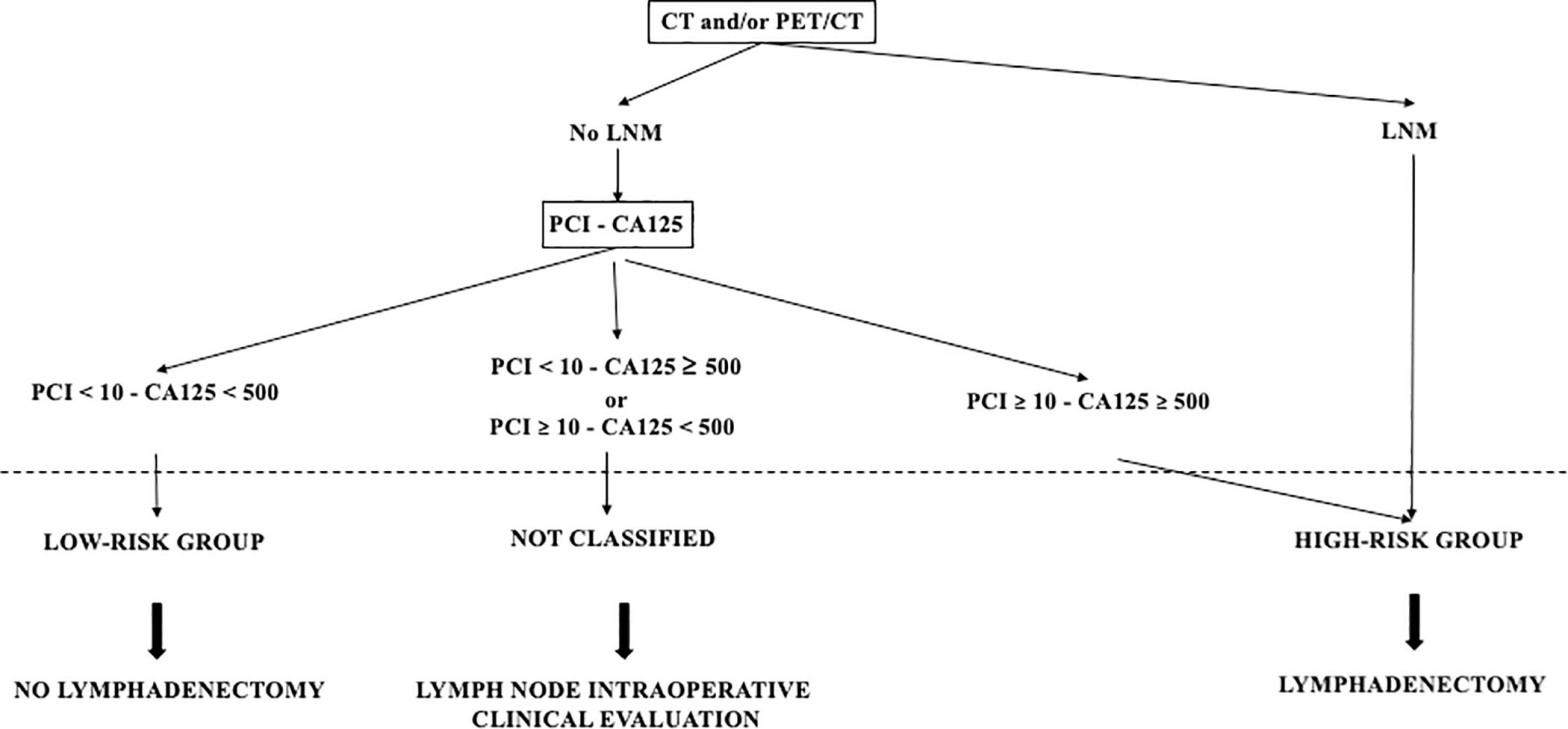
A decision tree of the simple lymphadenectomy decision rules.

**Fig 5:**
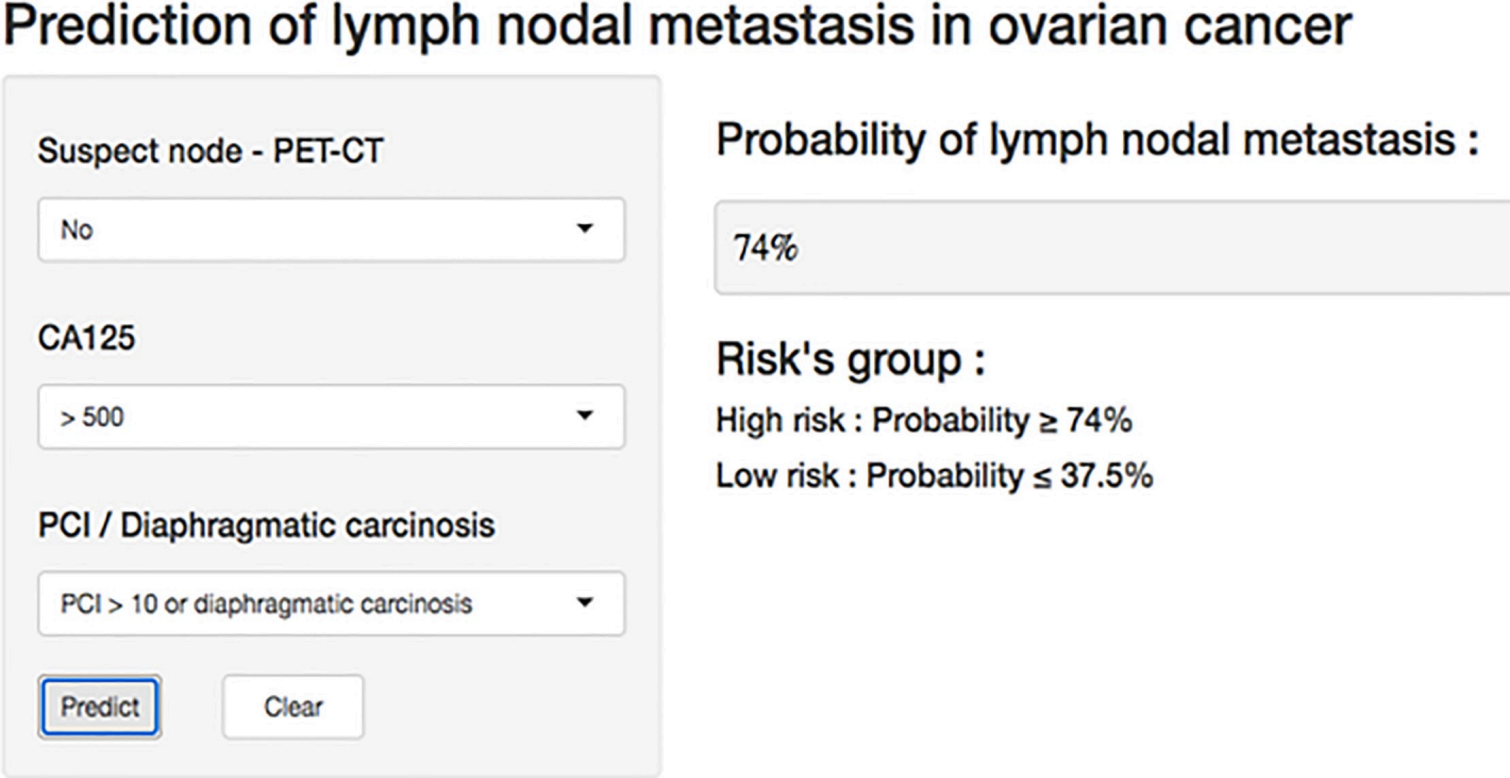
ShinyLNM.

Simple lymphadenectomy decision rules were proposed on the basis of these risk groups: patients in the low-risk group should not have lymphadenectomy whereas patients in the high-risk group should. Those rules are illustrated in a decision tree (Fig 4) and can also be easily used with our shinyLNM web app available at https://thomas-gaillard.shinyapps.io/Mimoun_node/. Fig 5 presents a sample screenshot.

## Discussion

We have constructed the first prediction model of pelvic and para-aortic LNM in patients with AEOC undergoing primary cytoreductive surgery. This study included 277 patients from the FRANCOGYN cohort: 115 with no LNM and 162 with LNM. The model was based on three pre-operative and intraoperative criteria: pelvic and/or para-aortic LNM on CT and/or PET/CT (aDOR = 5.02 95% CI [2.42–10.44], (p<0.00)), initial PCI ≥ 10 and/or diaphragmatic carcinosis (aDOR = 2.34 95% CI [1.13–4.83], p = 0.02), and initial CA125 ≥ 500 (aDOR = 2.03 95% CI [1.14–3.61], p = 0.02). There was no difference between the predicted and the observed probabilities of LNM (p = 0.09). Specificity for the group at high risk of LNM was 83.5%, the LR+ was 2.73, and the observed probability of LNM was 79.3%; sensitivity for the group at low-risk of LNM was 92.0%, the LR- was 0.24, and the observed probability of LNM was 25.0%.

Our study has several strengths. First, because the gold standard for the diagnosis of LNM was histology, misclassification bias was excluded. Moreover, we included only patients with at least 10 lymph nodes removed [9]. Specifically, we excluded patients with sampling of bulky nodes and selected only those with the lymphadenectomy dissection recommended for the cytoreductive surgery of every patient with AEOC before the publication of the LION trial in 2019. We then conducted an internal validation of the prediction model with the leave-one-out cross-validation procedure to correct for overoptimism [16]. Finally, two criteria of our prediction model have been described previously in the literature. In particular, an initial CA125 ≥ 500 has been associated with a higher rate of incomplete cytoreductive surgery [4] and an initial PCI < 10 corresponds to a complete cytoreductive surgery rate of 94% *vs* only 62% for an initial PCI ≥ 10 [19].

Two principal limitations of our study must be mentioned. The first is that we used a retrospective cohort to construct our prediction model, and collection bias may have occurred. Nonetheless, although this cohort is retrospective, it is also multicenter, with patients included from 11 French expert hospitals (FRANCOGYN group). This provided a large sample (277 patients) with good statistical power and tends to guarantee that our population is representative and that our results can be extrapolated. The second limitation is that there was no external validation with an independent sample but a second study with such a sample is planned for the very near future. Nonetheless, the internal validation may have enhanced the generalizability of the prediction model.

The publication of the LION trial in 2019 had a major impact on the surgical management of patients with AEOC who undergo primary cytoreductive surgery [6]. It is now clear that only patients with “suspect lymph nodes” at lymph node evaluation, preoperative imagery, or intraoperative clinical evaluation should have pelvic and/or paraaortic lymphadenectomy. Our study, consistent with those results, proposes a new more accurate tool for triaging patients according to simple lymphadenectomy decision rules:

- a patient in the group at low-risk of LMN (no LNM on CT and/or PET CT, PCI<10, CA125<500) should not have lymphadenectomy and the systematic opening of the retroperitoneal space for the intraoperative clinical evaluation should be omitted to reduce operative time and morbidity. Thus in our cohort, the false-negative rate for LNM was 25.0%, compared with 55.3% in the LION trial. Moreover, these 25.0% accounted for a mean of only 0.9 +/- 2.5 LNM among the 29.0 +/- 18.4 lymph nodes removed, while it has been proven that disease prognosis worsens with the number of LNMs removed [5, 9].
- a patient in the group at high-risk for LNM should have lymphadenectomy. In our cohort, the true-positive rate for LNM was 79.3% and not comparable to the LION trial. Moreover, in these 79.3% patients, among 36.5 +/- 16.0 lymph nodes removed, the mean number with LNM was 7.5 +/- 8.4. We note that in addition to the patients with LNM at CT and/or PET CT, already known to need lymphadenectomy, the high-risk group included a previously unknown category patient requiring lymphadenectomy—those with no LNM at CT and/or PET CT, CA125 ≥ 500, and PCI≥ 10. In this subgroup of 15 patients, 66.7% had LNM.
- patients not classified by the prediction model; for these patients, intraoperative clinical evaluation should still be performed. Our previous meta-analysis of the diagnostic accuracy of intraoperative clinical evaluation for detecting pelvic and para-aortic LNM in gynecological cancers, which included 5 studies and 723 patients, found a pooled specificity of 0.79, 95% CI [0.67–0.87], and was significantly higher in the subgroup of patients with only ovarian cancer: 0.92, 95% CI [0.85–0.98], with a pooled LR+ of 5.11, 95% CI [2.30–11.36]. Pooled sensitivity was 0.85, 95% CI [0.67–0.94] and pooled LR- was 0.25, 95% CI [0.16–0.38] [12].

In daily practice, surgeons can easily use these simple lymphadenectomy decision rules with the shinyLNM interactive web app to plan surgery appropriately and provide useful information to patients.

## Supporting information

S1 Dataset (XLSX)
